# Supplementary material for: Polyketide synthase gene clusters in the ericoid mycorrhizal fungus Oidiodendron maius: genome-wide prediction, expression profiling, and functional characterization of knockout mutants
Source: Front Plant Sci. 2026 Sep 16;17:1924430. doi: 10.3389/fpls.2026.1924430 (PMC13623903; doi:10.3389/fpls.2026.1924430)
Supplement: Supplementary file 1 [file SupplementaryFile1.zip › Supplementary Materials/Supplementary Folder 1.zip!/Oidma1_AssemblyScaffolds_Repeatmasked/knownclusterblast/cluster55/jgi.p_Oidma1_173701_mibig_hits.html]

| MIBiG Protein | Description | MIBiG Cluster | MiBiG Product | % ID | % Coverage | BLAST Score | E-value |
| --- | --- | --- | --- | --- | --- | --- | --- |
| EAL89345.1 | conserved\_hypothetical\_protein | BGC0001403 | Trypacidin\_biosynthetic\_gene\_cluster | 56 | 100.675675676 | 486 | 3.9e-137 |
| AGO59039.1 | PtaJ | BGC0000121 | Pestheic\_acid\_biosynthetic\_gene\_cluster | 53 | 99.7747747748 | 482 | 4.3e-136 |
| ADM34146.1 | hypothetical\_protein | BGC0001084 | Notoamide\_/\_stephacidin\_biosynthetic\_gene\_cluster | 48 | 99.0990990991 | 389 | 5e-108 |
| CBF90088.1 | conserved\_hypothetical\_protein | BGC0001271 | Emericellin\_biosynthetic\_gene\_cluster | 42 | 98.8738738739 | 351 | 1.5e-96 |
| AAS90020.1 | HypA | BGC0000007 | Aflatoxin\_biosynthetic\_gene\_cluster | 34 | 96.1711711712 | 233 | 3.5e-61 |
| AAS90113.1 | hypothetical\_protein | BGC0000006 | Aflatoxin\_biosynthetic\_gene\_cluster | 34 | 96.1711711712 | 232 | 7.8e-61 |
| AAS90039.1 | HypA | BGC0000008 | Aflatoxin\_biosynthetic\_gene\_cluster | 34 | 96.1711711712 | 230 | 3e-60 |
| AAS90089.1 | HypA | BGC0000010 | Aflatoxin\_biosynthetic\_gene\_cluster | 34 | 96.1711711712 | 230 | 3.9e-60 |
| BAE71335.1 | hypothetical\_protein | BGC0000004 | Aflatoxin\_biosynthetic\_gene\_cluster | 34 | 96.1711711712 | 226 | 5.6e-59 |
